# Prevalence and co-infection study of *Chlamydia trachomatis*, *Neisseria gonorrhoeae*, and *Trichomonas vaginalis* among symptomatic women using PCR assay

**DOI:** 10.1186/1471-2334-14-S3-P5

**Published:** 2014-05-27

**Authors:** SC Sonkar, K Wasnik, PK Mishra, P Mittal, A Kumar, J Suri, D Saluja

**Affiliations:** 1 Dr. B. R. Ambedkar Center for Biomedical Research, University of Delhi, Delhi-110007, India; 2 Department of Obstetrics & Gynecology Vardhman Mahavir Medical College and Safdarjung Hospital, New Delhi-110029, India

## Background

Sexually transmitted infections (STIs) are one of the major causes of acute illness, infertility, long term disability and death for millions of men, women and infants globally. *Trichomonas vaginalis*, *Neisseria gonorrhea* and *Chlamydia trachomatis* are well established agents of STIs leading to vaginal discharge in women. However, the prevalence and co infection patterns among symptomatic women is lacking in India. The present study aims to determine the prevalence and co infection of *Chlamydia trachomatis*, *Neisseria gonorrhoeae* and *Trichomonas vaginalis*, in women with vaginal discharge attending OPD, Vardhman Mahavir Medical College and Safdarjung Hospital, New Delhi.

## Methods

Dry swab samples were obtained and DNA was extracted used as template for PCR amplification using primers targeting *pfoB*, *gyr A* and *orf1* gene for diagnosis of *T. vaginalis*, *C. trachomatis* and *N. gonorrhoeae* respectively to estimate prevalence of co-infection.

## Results

A total of 335 women were studied (mean age 23.2 years), out of which 22 women (6.56 %) women were infected with had at least one pathogen. One woman (4.54 %) was co–infected with all three pathogens. Amongst 335 women, 18 women (5.37 %) tested positive for *T. vaginalis*, 02 women (0.59 %) tested positive for *C. trachomatis* and 1 woman (0.29 %) tested for *N. gonorrhea* by all the PCR assays, whereas 313 women (93.43 %) tested negative for the all three pathogens.

## Conclusion

The results demonstrated that it is necessary to test for all three pathogens namely *T. vaginalis*, *N. gonorrhea* and *C. trachomatis* in women with vaginal discharge.

